# Similarity-Based Predictive Models: Sensitivity Analysis and a Biological Application with Multi-Attributes

**DOI:** 10.3390/biology12070959

**Published:** 2023-07-04

**Authors:** Jeniffer D. Sanchez, Leandro C. Rêgo, Raydonal Ospina, Víctor Leiva, Christophe Chesneau, Cecilia Castro

**Affiliations:** 1 Department of Statistics and Applied Mathematics, Universidade Federal do Ceara, Fortaleza 60020-181, Brazil; jjduartes@dema.ufc.br (J.D.S.); leandro@dema.ufc.br (L.C.R.); 2 Department of Statistics, Universidade Federal de Pernambuco, Recife 50670-901, Brazil; raydonal@de.ufpe.br; 3 Department of Statistics, IME, Universidade Federal da Bahia, Salvador 40170-110, Brazil; 4 School of Industrial Engineering, Pontificia Universidad Católica de Valparaíso, Valparaíso 2362807, Chile; 5 Department of Mathematics, Université de Caen, 14032 Caen, France; christophe.chesneau@unicaen.fr; 6 Centre of Mathematics, Universidade do Minho, 4710-057 Braga, Portugal; cecilia@math.uminho.pt

**Keywords:** biological data, coefficient of variation, data science, distance measures, estimation methods, Monte Carlo simulation, predictive modeling, similarity functions

## Abstract

**Simple Summary:**

In this study, we perform a sensitivity analysis in similarity-based predictive models using computational simulations and two distinct methodologies, while focusing on a biological application. We utilize a linear regression model as a reference point. We gauge sensitivity by calculating the coefficient of variation of the parameter estimators from three different models. Our findings show that the first approach outperforms the second one when dealing with categorical variables. Moreover, this first approach offers the advantage of being more parsimonious due to a smaller number of parameters.

**Abstract:**

Predictive models based on empirical similarity are instrumental in biology and data science, where the premise is to measure the likeness of one observation with others in the same dataset. Biological datasets often encompass data that can be categorized. When using empirical similarity-based predictive models, two strategies for handling categorical covariates exist. The first strategy retains categorical covariates in their original form, applying distance measures and allocating weights to each covariate. In contrast, the second strategy creates binary variables, representing each variable level independently, and computes similarity measures solely through the Euclidean distance. This study performs a sensitivity analysis of these two strategies using computational simulations, and applies the results to a biological context. We use a linear regression model as a reference point, and consider two methods for estimating the model parameters, alongside exponential and fractional inverse similarity functions. The sensitivity is evaluated by determining the coefficient of variation of the parameter estimators across the three models as a measure of relative variability. Our results suggest that the first strategy excels over the second one in effectively dealing with categorical variables, and offers greater parsimony due to the use of fewer parameters.

## 1. Introduction

The empirical similarity prediction method does not assume a specific functional form relating the response variable to the covariates. Instead, it estimates the response variable value based on a weighted average of past response variable values, where the weights depend on the similarity of the covariate values. To apply empirical similarity in practice, a similarity measure and an estimation method are necessary.

Similarity measures are functions of distance that decrease as the distance decreases. They equal one when the distance is zero and converge to zero as the distance approaches infinity. The literature commonly considers two similarity measures: exponential inverse (EX) and fractional inverse (FR). These measures incorporate weighted distances, where the weights represent the relative importance of each covariate or level of a categorical variable. Estimating these weights from the data requires two methods: ordinary least squares (OLS) and maximum likelihood (ML).

The concept of empirical similarity has been axiomatized as a means to replicate human reasoning or natural behavior [1,2]. In [3], the identification, consistency, and distribution problems of the ML estimator for similarity models’ parameters were analyzed.

Categorical data, which includes multi-attribute records, are a crucial type of biological observations as they involve separable data and qualitative characteristics. Categorical data classify samples into mutually exclusive categories, often by counting the number of objects that fall into each qualitative class [4,5,6]. When dealing with categorical covariates, the empirical similarity literature within biological data describes two predictive approaches. The first approach, denoted as M1 and proposed in [7], maintains the categorical variables in their original formats. It assigns equal importance (weight) to all levels of the variable. The second approach, denoted as M2 and proposed in [8], encodes the categorical variables into binary variables, treating each category as a separate variable. In this case, different weights (influences) can be associated with each category of the same variable.

In predictive models, linear regression is a well-known and often used method. However, when dealing with categorical covariates, its utility can sometimes be limited. While linear regression provides a simple and interpretable model, it may not always capture the complexities of categorical covariates effectively. Therefore, alternative methods, such as empirical similarity models, may provide more nuanced and accurate predictions when dealing with such data types. Still, in our study, linear regression is utilized as a benchmark to provide a familiar frame of reference to readers and to aid comparison.

To the best of our knowledge, no previous studies have examined the sensitivity analysis of a specific class of similarity models concerning the accuracy of predicted values and the sensitivity of parameter estimators for the M1 and M2 methods. The choice of similarity and distance measures has been subjective in previous research. Thus, this study seeks to fill such a gap by performing a sensitivity analysis. Our study provides value by identifying which method yields the most robust predictions and parameter estimates under different scenarios.

Our main objective is to select similarity and distance measures that yield lower prediction errors and parameter estimators with reduced variability. The sensitivity of these models to environmental variations is simulated by splitting the data into training and test sets and calculating the coefficient of variation (CV) over multiple repetitions. The CV is a dimensionless and standardized measure of dispersion relative to the average of a dataset [9,10]. Given the different scales of the weights in the models, the CV is a suitable measure in our context.

To demonstrate the practical utility of our analysis, we employ a dataset on tooth length growth in Guinea pigs [11]. This dataset, involving different dosage levels and delivery methods of vitamin C, illustrates the models’ potential applications in biological research. The structure of the article is as follows: Section 2 provides a theoretical overview of empirical similarity and linear regression models. Section 3 describes the tooth length growth dataset used for simulating the sensitivity analysis. The methodology employed in the simulation study is detailed in Section 4. The results of the sensitivity analysis are presented in Section 5. In Section 6, our conclusion states a comparative analysis of M1, M2, and linear regression models, illustrating their competitive performances as gauged by the CV. We highlight the M1 method for its exceptional parsimony. The insights drawn from our research have potential to inform and guide researchers in selecting appropriate similarity and distance measures. Such informed selections can subsequently ensure predictions with enhanced accuracy and robustness in their parameter estimates.

## 2. Theoretical Background

In this study, we first introduce the linear regression model [12], as it serves like a benchmark for our detailed exploration of the performance of different predictive models under various scenarios. Consider a sample of size *n* with for the response variable, denoted as $Y_{1},\ldots ,Y_{n}$, which can be formulated as:

(1) $$Y_{i}=x_{i}^{⊤}w+\varepsilon_{i},\;i\in \left\{1,\ldots ,n\right\},$$

where $x_{i}^{⊤}=\left(x_{1i},\ldots ,x_{mi}\right)$ represents a 1×m vector of observed covariates, $w={\left(w_{1},\ldots ,w_{m}\right)}^{⊤}$ is an m×1 vector of weights for the regression model (fixed effects), and $\varepsilon_{i}$ denotes the model random error, with $\varepsilon_{i}∼N\left(0,\sigma^{2}\right)$. It is assumed that ${\left(\varepsilon_{1},\ldots ,\varepsilon_{n}\right)}^{⊤}$ are independent and identically distributed.

Let X be an n×m matrix with rank *m*, where each row represents $x_{i}^{⊤}$ (note that X is the known incidence matrix relating observations to fixed effects). Hence, $Y_{i}∼N\left(x_{i}^{⊤}w,\sigma^{2}\right)$, and the formulation stated in (1) represents a linear regression model [12,13].

The OLS estimator of w, which coincides with the ML estimator in this case, is given by:

(2) $$\hat{w}={\left(X^{⊤}X\right)}^{-1}X^{⊤}Y,$$

where $Y={\left(Y_{1},\ldots ,Y_{n}\right)}^{⊤}$. To predict a new observation $y_{t}$ with features $x_{t}^{⊤}=\left(x_{1t},\ldots ,x_{mt}\right)$, based on the model described in (1) and the estimate derived in (2), we use:

(3) $${\hat{y}}_{t}=\hat{y}\left(x_{t}\right)=x_{t}^{⊤}{\left(X^{⊤}X\right)}^{-1}X^{⊤}Y.$$

It is important to note that, assuming normality of errors, the variance of ${\hat{y}}_{t}$ in (3) can be calculated as:

$$\sigma^{2}x_{t}^{⊤}{\left(X^{⊤}X\right)}^{-1}x_{t}.$$

Now, we delve into the similarity model, considering the observations $\left(x_{1i},\ldots ,x_{mi},y_{i}\right)$, where $y_{i}$ represents the value of the random variable $Y_{i}$ for i∈{1,…,n}. We have a new vector of covariate values $x_{n+1}=\left(x_{1(n+1)},\ldots ,x_{m(n+1)}\right)$, and want to predict the future value of $Y_{n+1}$ as a weighted mean of the past values $y_{i}$. The weights depend on the similarity between the past features $x_{i}$ and the present value $x_{n+1}$ [1]. The similarity is measured by a function $s:R^{m}\times R^{m}\to R_{+}$. We provide a detailed explanation on how variations in the similarity function and other parameters impact the model’s performance.

Based on this concept, we give insights into the similarity model proposed in [8] and specify it as:

(4) $$Y_{t}=\frac{\sum_{i\neq t}s\left(x_{i},x_{t}\right)y_{i}}{\sum_{i\neq t}s\left(x_{i},x_{t}\right)}+\varepsilon_{t},\;1<t\leq n,$$

where the error term $\varepsilon_{i}$ represents a non-observable variable that accounts for the inherent uncertainty of the phenomenon under study, and $s(x_{i},x_{t})$ is a similarity measure between $x_{i}$ and $x_{t}$. Notably, the error term, for 1<t≤n, is uniquely defined as $\varepsilon_{1}=\sqrt{n}\left({\bar{Y}}_{n}-\alpha \right)$, where ${\bar{Y}}_{n}=\left(1/n\right)\sum_{i=1}^{n}Y_{i}$, and $\alpha =E(Y_{t})$. This special error term, $\varepsilon_{1}$ namely, is used to incorporate the inherent variability in the data that is not captured by the similarity measure $s(x_{i},x_{t})$. Such additional variability, as mentioned, might be due to the inherent uncertainty of the studied phenomenon or possible measurement errors. Moreover, $\varepsilon_{1}$ acts as a form of regularization, helping to avoid overfitting to the similarity model. This is particularly important for complex and high-dimensional models, where overfitting can be a relevant issue. Therefore, the specific need for $\varepsilon_{1}$, with 1<t≤n, arises to capture the additional variability in the data not addressed by the similarity measure and to provide regularization, avoiding overfitting.

In [8], parametric estimation of the similarity function, *s* say, was conducted. The estimation is considered parametric because $\varepsilon_{i}$ is assumed to follow a well-defined distribution with unknown parameters. The assumption is that the similarity function *s* is the same for all subjects generating $Y_{t}$ with t≤n. Two estimation methods are considered: OLS and ML.

Particularly, in [8], the study was focused on similarity functions that depend on a weighted Euclidean distance (WED). The square of the WED between two vectors $x=\left(x_{1},\ldots ,x_{m}\right)\in R^{m}$ and $x^{\prime }=\left(x_{1}^{\prime },\ldots ,x_{m}^{\prime }\right)\in R^{m}$ is defined as:

(5) $$d_{w}\left(x,x^{\prime }\right)=\sum_{j\leq m}w_{j}{\left(x_{j}-x_{j}^{\prime }\right)}^{2},$$

where $w=\left(w_{1},\ldots ,w_{m}\right)\in R_{+}^{m}$, as mentioned, represents a weight vector.

The function defined in (5) allows for different variables to have distinct influences on the distance measure, permitting adjustments for covariates with different scales. The weights in this function do not need to add up to one, providing flexibility in the modeling process. In the present study, we recall two specific similarity functions, denoted as EX and FR, are considered. These functions are defined as:

(6) $$s_{w}^{\mathrm{EX}}=e^{-d_{w}},\;s_{w}^{\mathrm{FR}}=\frac{1}{1+d_{w}},$$

where $s_{w}^{\mathrm{EX}}$ represents the exponential similarity function, and $s_{w}^{\mathrm{FR}}$ represents the fractional similarity function. These functions are derived from the WED, $d_{w}$ namely.

By incorporating these similarity functions into the model specified in (4), we obtain the parametric version of the empirical similarity model, which was estimated in [8] using the ML method. The ML estimation procedure is described in more detail in [7,8].

Utilizing the estimated values $\hat{w}$ obtained from the expression defined in (2) and the expressions given in (4), we can calculate the predicted value for a new observation $x_{t}$ using:

(7) $${\hat{Y}}_{t}=\frac{\sum_{i\neq t}\hat{s}\left(x_{i},x_{t}\right)y_{i}}{\sum_{i\neq t}\hat{s}\left(x_{i},x_{t}\right)},$$

where $\hat{s}$ represents the similarity function evaluated at $\hat{w}$.

In the case of handling categorical covariates, the distance measure defined in (5) is not suitable, particularly when there is no ordinal categories available. In such cases, a codification approach was proposed in [8], which involves transforming categorical variables into binary variables. This approach, referred to as M2, utilizes the WED stated in (5) to measure similarity. However, the method proposed in [8] has certain drawbacks. First, it may lead to a large number of parameters if a categorical variable has a high number of levels. Second, since different levels of the same categorical variable are treated as independent variables, they might be associated with significantly different weights, making the interpretation of the model more challenging. To address these issues, an alternative approach called M1 was introduced in [7] to handle categorical variables.

In the M1 approach, categorical variables are kept in their original format, and a weighted binary distance (WBD) is employed to measure similarity between vectors $x=\left(x_{1},\ldots ,x_{m}\right)\in R^{m}$ and $x^{\prime }=\left(x_{1}^{\prime },\ldots ,x_{m}^{\prime }\right)\in R^{m}$. The WBD is defined as:

(8) $$d_{w}\left(x,x^{\prime }\right)=\sum_{l\leq m}w_{l}1_{l}\left(x_{l},x_{l}^{\prime }\right),$$

where $1_{l}\left(x_{l},x_{l}^{\prime }\right)$ is an indicator function given by:

$$1_{l}\left(x_{l},x_{l}^{\prime }\right)=\left\{\begin{array}{cc} 0, & \mathrm{if}\;x_{l}=x_{l}^{\prime },\\ 1, & \mathrm{if}\;x_{l}\neq x_{l}^{\prime }. \end{array}\right.$$

Thus, the WBD stated in (8) sums the weights associated with covariates that have different observed values. Consequently, the predicted value for the response variable related to a given set of features is obtained as the weighted mean of the other observed values of this variable, where observations with more features in common in relation to the given set have a higher weight.

Here, we explore the use of the weighted Minkowski distance (WMD) to handle dichotomous covariates, considering ordinal categories. The WMD of order γ between two vectors $x=\left(x_{1},\ldots ,x_{m}\right)\in R^{m}$ and $x^{\prime }=\left(x_{1}^{\prime },\ldots ,x_{m}^{\prime }\right)\in R^{m}$ is defined as:

(9) $$d_{w}^{\mathrm{WMD}}\left(x,x^{\prime }\right)={\left(\sum_{l=1}^{m}w_{l}{\left|x_{l}-x_{l}^{\prime }\right|}^{\gamma }\right)}^{1/\gamma }.$$

Since the WMD stated in (9) introduces an additional parameter γ, we also introduce another parameter δ in the WBD and WED to provide more flexibility in explaining the observed data. The WBD and the WED are then stated as:

(10) $$d_{w,\delta }^{\mathrm{WBD}}\left(x,x^{\prime }\right)={\left(\sum_{l\leq m}w_{l}I_{l}\left(x_{l}-x_{l}^{\prime }\right)\right)}^{\delta },\;d_{w,\delta }^{\mathrm{WED}}\left(x,x^{\prime }\right)={\left(\sum_{l\leq m}w_{l}{\left(x_{l}-x_{l}^{\prime }\right)}^{2}\right)}^{\delta }.$$

Note that, as δ increases, the distances defined in (10) also increase. However, for δ=1, we obtain the standard distance measures, $d_{w,1}^{\mathrm{WBD}}=d_{w}^{\mathrm{WBD}}$. The approach that handles categorical covariates without codification is M1. It is important to emphasize that for M2, where all covariates are binary, the Minkowski, binary, and Euclidean distances coincide.

## 3. Biological Application

The biological dataset used in this study investigates the effect of vitamin C on the tooth growth of Guinea pigs. Scientifically known as *Cavia porcellus*, Guinea pigs are rodents belonging to the *Caviidae* family and the *Cavia* genus [14]. The dataset consists of 60 observations, where the response variable (*Y*) is the length of the Guinea pig tooth measured in micrometers (μm), and the covariates are as follows:

- Vitamin C dose ($X_{1}$): This covariate is measured in milligrams (mg) and has three levels: 0.5 mg, 1.0 mg, and 2.0 mg. The vitamin C dose variable is ordinal.
- Food supplemental type ($X_{2}$): This covariate has two categories: ascorbic acid (VC) and orange juice (OJ). These categories are represented as 0 and 1, respectively. The food supplemental type variable is also ordinal.

**Figure 1:**
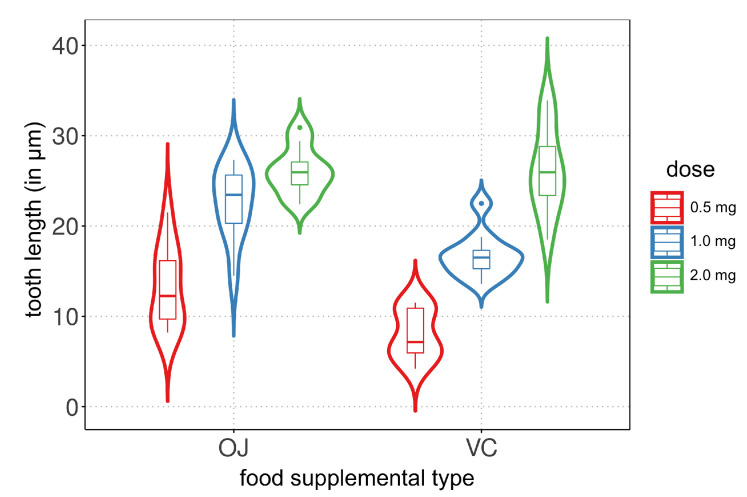
Violin plots of tooth length (in μm) for listed vitamin C dose (in mg) and food supplement.

To conduct an exploratory data analysis, violin plots are created to visualize the tooth length distribution based on the vitamin C dose and food supplemental type. Figure 1 shows the violin plots, where each plot represents the distribution of tooth length for a specific combination of the two covariates. The plots reveal that the vitamin C dose has an impact on tooth growth, showing a similar trend in both food supplemental types. However, there are differences in the central tendency and variability measures between the two types. A violin plot combines the features of a box plot and a kernel density plot, providing information about the data distribution and density peaks.

## 4. Setup to Evaluate Sensitivity

In the Monte Carlo simulation, a sensitivity analysis was conducted to assess the variability of the parameter estimators and the predicted values of the response variable. The simulation consisted of 30 iterations, where each iteration involved a randomly generated training dataset comprising 70% of the total data, and a test dataset comprising the remaining 30%.

For each training dataset, numerical computations were performed to obtain parameter estimates using the empirical similarity methods. To initiate the estimation process, five initial parameter values were considered. The specific values of these initial parameters are not provided in the given text and should be defined based on the methodology and requirements of the empirical similarity methods used in the study as:

- For each fixed value of vitamin C dose (0.5, 1.0 and 2.0), the mean of the response variable is calculated. Let us denote these means by ${\bar{y}}_{11}$, ${\bar{y}}_{12}$ and ${\bar{y}}_{13}$, respectively.
- For each fixed value of the supplemental type (VC and OJ), the mean of the response variable is also computed and denoted by ${\bar{y}}_{21}$ and ${\bar{y}}_{22}$, respectively.
- The five initial parameter values $\left(w_{1}^{0},w_{2}^{0}\right)$ for M1 are: $({\bar{y}}_{11},{\bar{y}}_{21}),$$({\bar{y}}_{11},{\bar{y}}_{22}),$$\left({\bar{y}}_{12},{\bar{y}}_{21}\right)$, $\left({\bar{y}}_{12},{\bar{y}}_{22}\right)$, and $\left({\bar{y}}_{13},{\bar{y}}_{21}\right)$.
- The five initial parameter values $\left(w_{1}^{0},w_{2}^{0},w_{3}^{0},w_{4}^{0}\right)$ for M2 are: $({\bar{y}}_{11},{\bar{y}}_{11},{\bar{y}}_{11},{\bar{y}}_{21}),$$({\bar{y}}_{11},{\bar{y}}_{11},$${\bar{y}}_{11},{\bar{y}}_{22})$, $\left({\bar{y}}_{12},{\bar{y}}_{12},{\bar{y}}_{12},{\bar{y}}_{21}\right)$, $\left({\bar{y}}_{12},{\bar{y}}_{12},{\bar{y}}_{12},{\bar{y}}_{22}\right)$, and $\left({\bar{y}}_{13},{\bar{y}}_{13},{\bar{y}}_{13},{\bar{y}}_{21}\right)$.

For each of the initial parameter values, the model is estimated for the training data and the mean square error (MSE) for the prediction in the test data is calculated. The predicted response value and the estimated weights for the case with a minimal MSE are chosen.

We investigated the variability by modifying the following aspects:

- Models: M1 [7], M2 [8], and the linear regression [12].
- Estimation methods: ML and OLS.
- Similarity functions: EX and FR.
- Distance measures: WBD, WED, and WMD for M1; and WED for M2. We test the values {1/4,1/2,1,2,4} for the parameter γ of the WMD, and the values {1,2,4} for the parameter δ in the modified WBD and WED. Values of δ less than one are also tested, but they do not provide convergence in the estimation algorithm.

To quantify the variability of the parameter estimators and the predicted response variable, the empirical CV and MSE are calculated based on the 30 iterations of the Monte Carlo simulation.

For M1:

- The parameter estimators correspond to $w_{1}$ (intercept) and $w_{2}$ (associated with an increase in dose of 1.0 mg).
- The empirical CV of the parameter estimators can be calculated as the ratio of the sample standard deviation to the sample mean of the parameter estimates $w_{1}$ and $w_{2}$ across the 30 iterations.
- The MSE of the predicted response variable may be computed as the average squared difference between the predicted response variable values and the true values across the 30 iterations.

For M2:

- The parameter estimators correspond to $w_{1}$ (intercept), $w_{2}$ (associated with dose of 0.5 mg), $w_{3}$ (associated with dose of 1.0 mg), and $w_{4}$ (associated with dose of 2.0 mg and supplemental type).
- The empirical CV of the parameter estimators can be obtained as the ratio of the sample standard deviation to the sample mean of the parameter estimates $w_{1}$, $w_{2}$, $w_{3}$, and $w_{4}$ across the 30 iterations.
- The MSE of the predicted response variable can be determined as the average squared difference between the predicted response variable values and the true values across the 30 iterations.

## 5. Simulation Results

The simulations were carried out on a computer equipped with an Intel© Core™, i7-5500UK CPU 4 gigahertz, 16 gigabyte RAM, System Type 64 bit operating system Linux, using the R language, a software environment for statistical computing and graphics, in its version 3.5.2 [15]. Codes are available upon request from the authors.

Based on our simulation results, it is clear that the empirical similarity models (M1 and M2) and the linear regression model show comparable performance in terms of the mean MSE of the predicted values for the length of the Guinea pig tooth, a testament to the robustness of our analysis.

While we recognize that an increased sample size might result in a broader distribution of the results, the consistent findings among different models under our current conditions attest to the reliability of our work. Furthermore, our chosen sample size reflects a practical balance between computational complexity and statistical validity, a key consideration in all real-world application.

**Figure 2:**
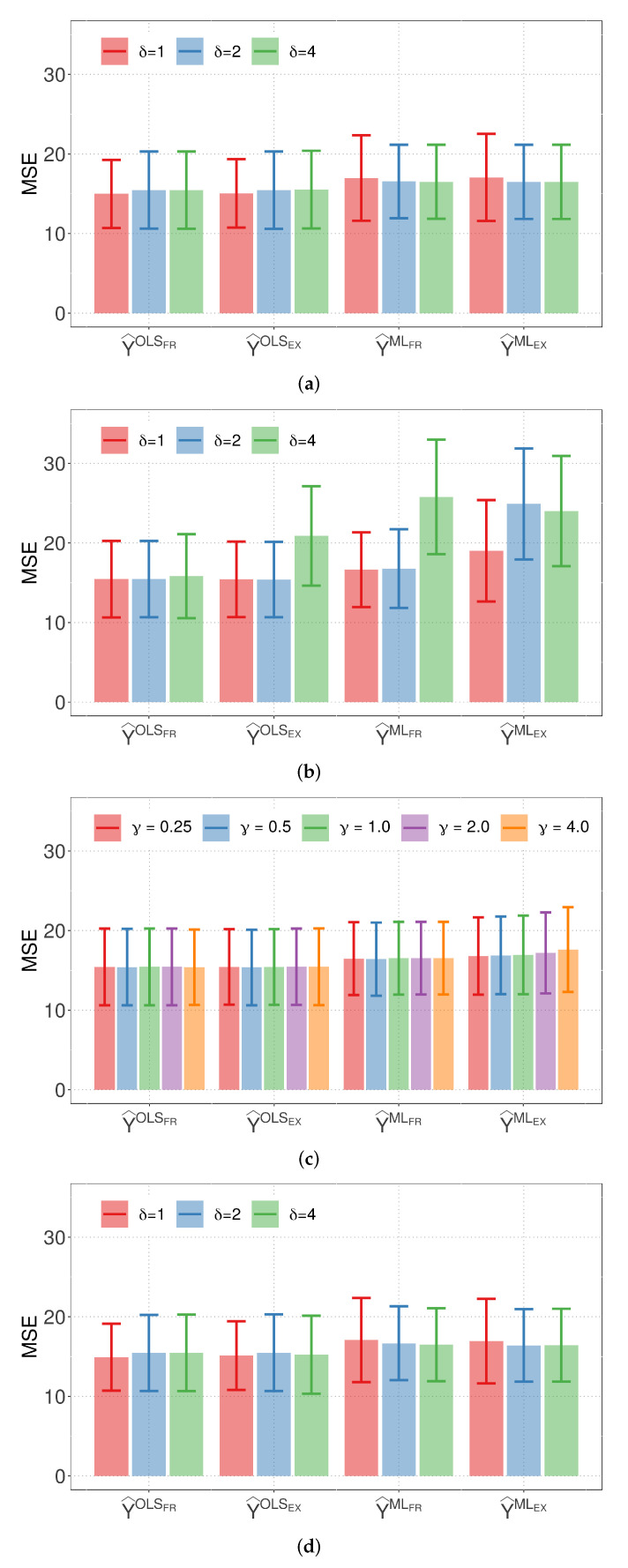
Plots of average MSE of the indicated response prediction and parameter (with error bars) for: (**a**) model M1 and binary distance; (**b**) Model M1 and Euclidean distance; (**c**) Model M1 and Minkowski distance; and (**d**) model M2 and Euclidean distance.

The mean MSE and standard deviation of the linear regression model, being comparable to those of M1 and M2, serve as a strong benchmark in our analysis. Furthermore, from Figure 2, we find no significant statistical difference among the MSE of the response variable predictions for all tested models, in addition to corroborating the robustness of our chosen models. Such robustness underlines the adaptability of these models to different scenarios and conditions. It serves as a valuable insight for making informed decisions on model selection, considering factors such as model complexity, interpretability, and specific objectives of the analysis. Notably, M1 stands out as the most parsimonious model, requiring only two parameters. This parsimony enhances its applicability, particularly when dealing with a large number of categorical covariates or when these covariates have numerous levels.

In summary, the empirical similarity models (M1 and M2) and the linear regression model demonstrate competitive performance in terms of the variability of the predicted values for the length of the Guinea pig tooth. These insights from the simulation and data analysis can guide the anticipation of the models’ performance under different conditions and make adjustments to the research design accordingly. Our findings contribute to the current literature on empirical similarity prediction models, and we consider further research with larger Monte Carlo simulations and other comparison strategies in the future.

**Figure 3:**
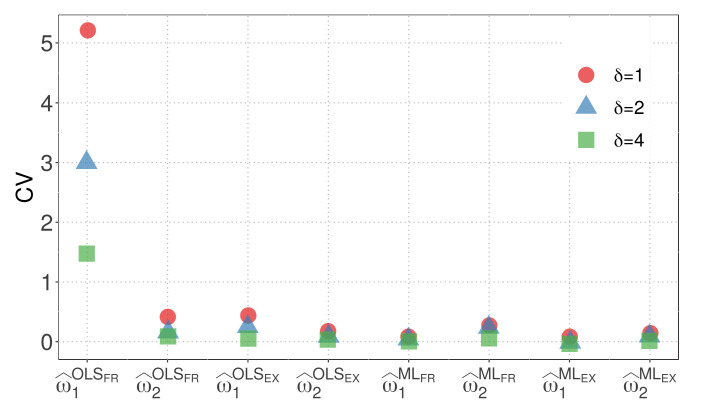
CVs of parameter estimators for model M1 with WBD.

**Figure 4:**
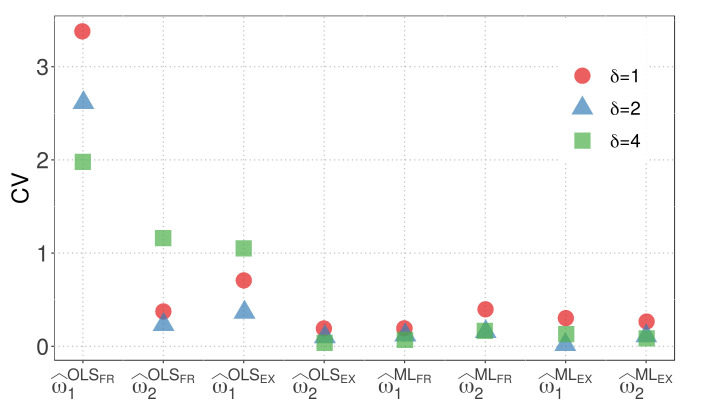
CVs of parameter estimators for model M1 with WED.

Figure 3 and Figure 4 display the CVs of the parameter estimators for model M1 when the WBD and WED are considered, respectively. The plots illustrate the impact of different similarity measures and estimation methods on the variability of the parameter estimators. The results highlight the influence of the exponential inverse similarity and the choice of the ML method, particularly when δ=4, in reducing the CVs for the parameter estimates.

Figure 3 and Figure 4 provides valuable insights, summarized as follows:

- The OLS method, when used with the fractional inverse similarity, exhibits high variability in the estimates for the parameter $w_{1}$.
- Increasing the value of the parameter δ results in parameter estimates with lower variability.
- The exponential inverse similarity generally produces parameter estimates with less variability compared to the fractional inverse similarity.
- The ML method generally provides estimates with lower variability for the parameter $w_{1}$ compared to the OLS method.
- The minimum CVs obtained for ${\hat{w}}_{1}$ and ${\hat{w}}_{2}$ are 0.01 and 0.05, respectively, when the ML method is utilized with the exponential inverse similarity and δ=4.

**Figure 5:**
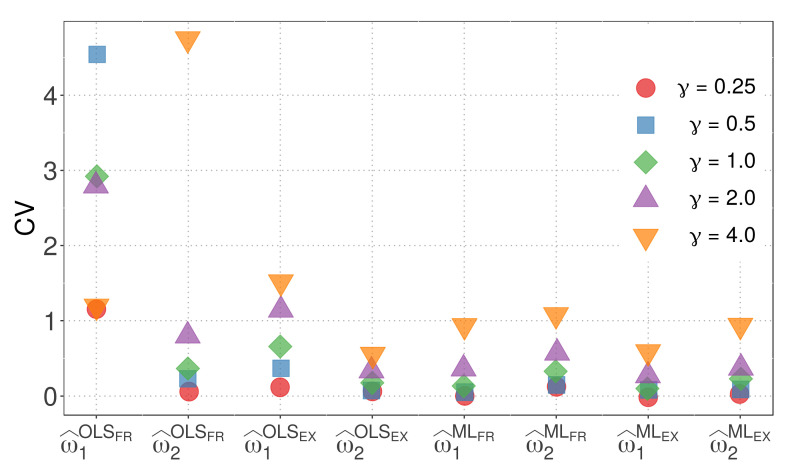
CVs of the parameter estimators for model M1with WMD.

By combining these insights, we gain a comprehensive understanding of the variability in the parameter estimators for model M1 with different similarity measures and distances. Figure 5 illustrates the CVs of the parameter estimators for model M1 when the WMD is employed.

The following observations can be made from Figure 5:

- The OLS method, in conjunction with the FR similarity, yields parameter estimates with notably high variability.
- With the exception of the parameter ${\hat{w}}_{1}$ estimated using the OLS method and FR similarity, the variability of parameter estimates generally increases with higher values of γ.
- The EX similarity measure, except for the case of ${\hat{w}}_{1}$ estimated using the OLS method and γ=4, results in parameter estimates with less variability than the FR similarity.
- In all other cases, parameter estimates obtained using the ML method exhibit less variability compared to the corresponding estimates from the OLS method.
- The combination of the ML method with the EX similarity and γ=1/4 yields the lowest variability, as evidenced by the sum of the CVs of ${\hat{w}}_{1}$ and ${\hat{w}}_{2}$, which are 0.03 and 0.07, respectively.

Figure 5 provides valuable insights into the variability of parameter estimators for model M1 with different similarity measures and the Minkowski distance. It is evident that the choice of similarity measure and estimation method can significantly impact the variability of the parameter estimators. Furthermore, the ML method, particularly when used with the EX similarity and appropriate parameter values, demonstrates superior performance in terms of reduced variability.

**Figure 6:**
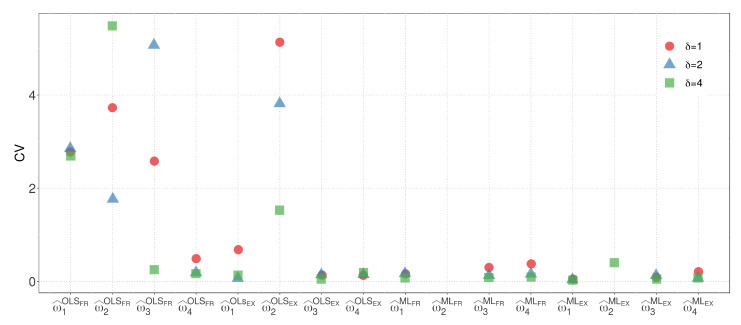
CVs of the parameter estimators for model M2.

**Figure 7:**
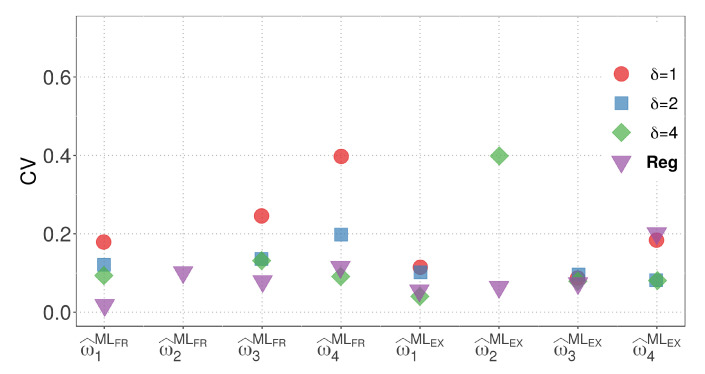
CVs of the parameter estimators for the regression model using the ML method.

By considering the results from both models, we can compare the variability of the parameter estimates for M1 and M2 with the regression model. Model M2, which has the same number of parameters as the regression model, allows for a more straightforward visual comparison. In terms of the EX similarity, except for the case where δ=4, where the estimate of the weight $w_{2}$ has high variability, the estimates for model M2 exhibit competitive variability with the regression model in other cases. Figure 6 and Figure 7 present a visualization of the CVs of the parameter estimates for model M2 and the regression model using the ML method, respectively. The figures highlight the variability of the estimates and show the competitive performance of model M2, with the EX similarity yielding consistent results except for the case where δ=4.

From Figure 6 and Figure 7, the following observations can be stated:

- The OLS method, when used with the FR similarity, yields parameter estimates for $w_{1}$, $w_{2}$, and $w_{3}$ with high variability. Additionally, when the EX similarity is employed, the estimates for $w_{2}$ exhibit increased variability.
- Estimates of parameter $w_{2}$ are consistently zero when the ML method is utilized with δ=1 and δ=2, indicating that the variable dose of 1.0 mg has no influence on the response variable estimation.
- Among the tested scenarios, using δ=4 results in estimates with the least variability in 13 out of 16 cases.
- The EX similarity measure consistently provides parameter estimates with variability at least as low as, if not lower than, those obtained with the FR similarity in 20 out of 24 cases.
- When considering the sum of the CVs of ${\hat{w}}_{1}$, ${\hat{w}}_{2}$, ${\hat{w}}_{3}$, and ${\hat{w}}_{4}$, the combination of the ML method with the EX similarity and δ=2 yields the lowest variability. In this case, the CVs of ${\hat{w}}_{1}$, ${\hat{w}}_{2}$, ${\hat{w}}_{3}$, and ${\hat{w}}_{4}$ are 0.07, 0.00, 0.08, and 0.11, respectively.

When comparing the M1, M2, and regression models, M2 stands out as the most suitable for visual comparison due to its equal number of parameters. Based on minimal CV, we selected model M2 due to its best fit. Figure 6 and Figure 7 also compare the regression and M2 models using the ML method, consistently showing superior results for model M2. While the estimate of weight $w_{2}$ exhibits higher variability when δ=4 in the EX similarity case, the variability of the estimates for model M2 remains competitive with that of the regression model in the other cases.

## 6. Conclusions

This study aimed to evaluate the performance of empirical similarity models in a biological application, specifically in the context of predicting the length of Guinea pig teeth. Two empirical similarity models, M1 and M2, were compared against a linear regression model, serving as a benchmark. On the one hand, M1 preserved the original format of categorical covariates and utilized general distance measures with a single weight assigned to each covariate. On the other hand, M2 constructed binary variables for each level of the categorical covariates and employed similarity measures based solely on the Euclidean distance. For both M1 and M2, parameter estimation was conducted using ordinary least squares and maximum likelihood methods. It was observed that the maximum likelihood method consistently provided parameter estimates with low variability across both models, emphasizing the robustness of our approach.

In terms of the mean square error of the predicted response values, all models demonstrated competitive performance. Interestingly, M1 emerged as the most parsimonious model with only two parameters. The mean square error of the predicted values for the empirical similarity models did not exhibit dependency on the estimation method, similarity function, or distance measure. Different similarity functions were also explored for both models, including the exponential inverse and fractional inverse similarity functions.

The results indicated that the exponential inverse similarity function yielded less variability in most scenarios. For M1, three different distance functions were tested: weighted binary, Euclidean, and Minkowski distances. In the case of the binary and Euclidean distances, introducing an exponential parameter (δ∈{1,2,4}) reduced variability. For the Minkowski distance, smaller values of the parameter γ resulted in better performance. However, it is important to note that values of δ greater than four or values of γ less than 1/4 may lead to convergence issues in the estimation algorithms. In our analysis, when we employed the maximum likelihood estimation and the exponential inverse similarity function, we observed that the coefficients of variation for the parameter estimates were similar across the M1, M2, and linear regression models. This suggests that such models are comparable in terms of sensitivity. Nonetheless, M1 stands out due to its simplicity, requiring only two parameters. This simplicity can be particularly advantageous when dealing with a large number of categorical covariates or when these covariates have numerous levels.

To address potential overfitting in the M2 model, regularization techniques could be introduced [16]. However, to ensure a fair comparison between the models, we did not introduce this penalty in the empirical similarity framework in the current study. Nevertheless, we recognize the value of incorporating regularization methods in future investigations to explore their impact on the performance and generalization ability of the empirical similarity models.

It is important to acknowledge that the performance of these models can be influenced by several factors that warrant further investigation. Among these factors are the total sample size, the distribution of samples across different covariate categories, and the number of simulations performed. Although our findings contribute significantly to the existing literature on empirical similarity prediction models and methods, we deem it crucial to conduct expanded research to explore and understand the potential impact of these factors. Furthermore, the utility of simulation and data analysis is evident in our study. They serve as strategic tools, providing a clear understanding of how the models perform under different scenarios. Such insights permit informed adjustments to the models, thereby enhancing their functionality. The comprehensive analysis of model performance, encompassing different scenarios, similarity functions, and distance measures, facilitates effective decisions regarding model selection, taking into account specific objectives of the analysis, complexity, and interpretability.

In sum, our work contributes significantly to the burgeoning field of empirical similarity prediction models and methods. Our findings, which provide robust models capable of handling a variety of scenarios, serve as a foundation for future research, particularly for further exploration of the impact of sample size, number of simulations, and distribution of covariates on the performance of these models, as mentioned.

## Data Availability

The data and codes are available upon request.

## References

[1] Gilboa I., Lieberman O., Schmeidler D. (2006). Empirical similarity. Rev. Econ. Stat..

[2] Raza B., Kumar Y.J., Malik A.K., Anjum A., Faheem M. (2018). Performance prediction and adaptation for dataset management system workload using case-based reasoning approach. Inf. Syst..

[3] Lieberman O. (2010). Asymptotic theory for empirical similarity models. Econom. Theory.

[4] Xu B., Feng X., Burdine R.D. (2010). Categorical data analysis in experimental biology. Dev. Biol..

[5] Mayya S.S., Monteiro A.D., Ganapathy S. (2017). Types of biological variables. J. Thorac. Dis..

[6] Larrabee B., Scott H.M., Bello N.M. (2014). Ordinary least squares regression of ordered categorical data: Inferential implications for practice. J. Agric. Biol. Environ. Stat..

[7] Sanchez J.D., Rêgo L.C., Ospina R. (2019). Prediction by empirical similarity via categorical regressors. Mach. Learn. Knowl. Extr..

[8] Gayer G., Gilboa I., Lieberman O. (2007). Rule-based and case-based reasoning in housing prices. B.E. J. Theor. Econ..

[9] Riquelme M., Leiva V., Galea M., Sanhueza A. (2011). Influence diagnostics on the coefficient of variation of elliptically contoured distributions. J. Appl. Stat..

[10] Ospina R., Marmolejo-Ramos F. (2019). Performance of some estimators of relative variability. Front. Appl. Math. Stat..

[11] De Miguel C., Saniotis A., Cieslik A., Henneberg M. (2022). Comparative study of brain size ontogeny: Marsupials and placental mammals. Biology.

[12] Bucchi A., Del Bove A., López-Lázaro S., Quevedo-Díaz F., Fonseca G.M. (2022). Digital reconstructions using linear regression: How well can it estimate missing shape data from small damaged areas?. Biology.

[13] Judge G.G., Griffiths W.E., Hill C., Lee T.C. (1985). The Theory and Practice of Econometrics.

[14] Crampton E. (1947). The growth of the odontoblasts of the incisor tooth as a criterion of the vitamin C intake of the Guinea pig. J. Nutr..

[15] R Core Team (2021). R: A Language and Environment for Statistical Computing.

[16] Tutz G., Gertheiss J. (2016). Regularized regression for categorical data. Stat. Model..

